# Supplementary material for: Frequent sgRNA-barcode recombination in single-cell perturbation assays
Source: PLoS One. 2018 Jun 6;13(6):e0198635. doi: 10.1371/journal.pone.0198635 (PMC5991360; doi:10.1371/journal.pone.0198635)
Supplement: S1 Table — (DOCX) [file pone.0198635.s001.docx]

**S1 Table. Primers used in this study**

| Name | Description | Sequence |
| --- | --- | --- |
| PCR1 + | Sense primer for PCR1, land on U6 promoter | GACTATCATATGCTTACCGT |
| PCR1 - | Antisense primer for PCR2, next to barcode | GTACAAGCAAAAAGCAGATCTTGTCTTCGTTGGGAGTG |
| P5 Barlib stag 1 | PCR2 primer for Barcode library prep, P5 end, with stager sequence | AATGATACGGCGACCACCGAGATCTACACTCTTTCCCTACACGACGCTCTTCCGATCTCCGCCTCCCCGCTGTACA |
| P5 Barlib stag 2 | PCR2 primer for Barcode library prep, P5 end, with stager sequence | AATGATACGGCGACCACCGAGATCTACACTCTTTCCCTACACGACGCTCTTCCGATCTACCGCCTCCCCGCTGTACA |
| P5 Barlib stag 3 | PCR2 primer for Barcode library prep, P5 end, with stager sequence | AATGATACGGCGACCACCGAGATCTACACTCTTTCCCTACACGACGCTCTTCCGATCTTACCGCCTCCCCGCTGTACA |
| P5 Barlib stag 4 | PCR2 primer for Barcode library prep, P5 end, with stager sequence | AATGATACGGCGACCACCGAGATCTACACTCTTTCCCTACACGACGCTCTTCCGATCTGTTCCGCCTCCCCGCTGTACA |
| P5 Barlib stag 5 | PCR2 primer for Barcode library prep, P5 end, with stager sequence | AATGATACGGCGACCACCGAGATCTACACTCTTTCCCTACACGACGCTCTTCCGATCTTGTACCGCCTCCCCGCTGTACA |
| P7 Barlib N720 | PCR2 primer for Barcode lib prep, P7 end | CAAGCAGAAGACGGCATACGAGATAGGCTCCGGTGACTGGAGTTCAGACGTGTGCTCTTCCGATCTTTGGCCTAGCTCTAAAAC |
| P7 Barlib N721 | PCR2 primer for Barcode lib prep, P7 end | CAAGCAGAAGACGGCATACGAGATGCAGCGTAGTGACTGGAGTTCAGACGTGTGCTCTTCCGATCTTTGGCCTAGCTCTAAAAC |
| P7 Barlib N722 | PCR2 primer for Barcode lib prep, P7 end | CAAGCAGAAGACGGCATACGAGATCTGCGCATGTGACTGGAGTTCAGACGTGTGCTCTTCCGATCTTTGGCCTAGCTCTAAAAC |
| P7 Barlib N723 | PCR2 primer for Barcode lib prep, P7 end | CAAGCAGAAGACGGCATACGAGATGAGCGCTAGTGACTGGAGTTCAGACGTGTGCTCTTCCGATCTTTGGCCTAGCTCTAAAAC |
| P7 Barlib N724 | PCR2 primer for Barcode lib prep, P7 end | CAAGCAGAAGACGGCATACGAGATCGCTCAGTGTGACTGGAGTTCAGACGTGTGCTCTTCCGATCTTTGGCCTAGCTCTAAAAC |
| P7 Barlib N726 | PCR2 primer for Barcode lib prep, P7 end | CAAGCAGAAGACGGCATACGAGATGTCTTAGGGTGACTGGAGTTCAGACGTGTGCTCTTCCGATCTTTGGCCTAGCTCTAAAAC |

**Color Note:**

Green: P5 / P7 adapter for flowcell clustering.

Blue: illumina sequencing primer binding site.

Red: sequence on the amplicon.

Orange: P7 index.

Purple: insertion.
